# Musculoskeletal impairment survey in Rwanda: Design of survey tool, survey methodology, and results of the pilot study (a cross sectional survey)

**DOI:** 10.1186/1471-2474-8-30

**Published:** 2007-03-28

**Authors:** Oluwarantimi Atijosan, Hannah Kuper, Dorothea Rischewski, Victoria Simms, Christopher Lavy

**Affiliations:** 1 Trauma Unit, John Radcliffe Hospital, Oxford, OX3 9DU, UK.; 2 International Centre Of Eye Health, London School Of Hygiene & Tropical Medicine, Keppel Street, WC1E 7HT London. UK.; 3 Palliative Care, Policy and Rehabilitation, King's College London, London SE5 9RJ, UK.; 4 Nuffield Department of Orthopaedic Surgery, University Of Oxford, Windmill Road, Oxford OX3 7LD, UK.

## Abstract

**Background:**

Musculoskeletal impairment (MSI) is an important cause of morbidity and mortality worldwide, especially in developing countries. Prevalence studies for MSI in the developing world have used varying methodologies and are seldom directly comparable. This study aimed to develop a new tool to screen for and diagnose MSI and to pilot test the methodology for a national survey in Rwanda.

**Methods:**

A 7 question screening tool to identify cases of MSI was developed through literature review and discussions with healthcare professionals. To validate the tool, trained rehabilitation technicians screened 93 previously identified gold standard 'cases' and 86 'non cases'. Sensitivity, specificity and positive predictive value were calculated. A standardised examination protocol was developed to determine the aetiology and diagnosis of MSI for those who fail the screening test. For the national survey in Rwanda, multistage cluster random sampling, with probability proportional to size procedures will be used for selection of a cross-sectional, nationally representative sample of the population. Households to be surveyed will be chosen through compact segment sampling and all individuals within chosen households will be screened. A pilot survey of 680 individuals was conducted using the protocol.

**Results:**

The screening tool demonstrated 99% sensitivity and 97% specificity for MSI, and a positive predictive value of 98%. During the pilot study 468 out of 680 eligible subjects (69%) were screened. 45 diagnoses were identified in 38 persons who were cases of MSI. The subjects were grouped into categories based on diagnostic subgroups of congenital (1), traumatic (17), infective (2) neurological (6) and other acquired(19). They were also separated into mild (42.1%), moderate (42.1%) and severe (15.8%) cases, using an operational definition derived from the World Health Organisation's International Classification of Functioning, Disability and Health.

**Conclusion:**

The screening tool had good sensitivity and specificity and was appropriate for use in a national survey. The pilot study showed that the survey protocol was appropriate for measuring the prevalence of MSI in Rwanda. This survey is an important step to building a sound epidemiological understanding of MSI, to enable appropriate health service planning.

## Background

Musculoskeletal impairment (MSI) is an important cause of morbidity and mortality worldwide, [1] however the exact magnitude of the global burden of disease due to MSI is unknown. The World Health Organisation (WHO) estimated that 10% of any population has a disability, of which 1.5% requires rehabilitation[2]. WHO also estimated that there is a disproportionate burden of MSI borne by the developing world[3,4] as 80% of people living with disabilities live in developing countries and many do not have access to rehabilitation services.

There have been few surveys conducted to assess the magnitude and causes of MSI in developing countries and these have used a variety of different methodologies [5-12] so the results from the surveys are not easily comparable. The majority of studies look at disability as a whole, rather than physical disability or MSI. Some of these surveys have focused on the total population, while others have focussed on children or other age groups. Most surveys have been undertaken in districts or communities rather than on a national level.

Rwanda is a poor country in central Africa, with a population of around 8.2 million people in 2002 [13]. Rwanda suffered a major internal war in 1994 during which up to one million people were killed. In 1995, Handicap International carried out an all-age nationwide survey in Rwanda and estimated that the prevalence of disability was 0.58%, of which physical deformities were the main cause [Enquête Nationale sur l'Ampleur du Handicap au Rwanda : Résultats et recommandations pour l'élaboration d'un plan. In. Kigali: Handicap International, Ministère de la Réhabilitation et l'Intégration Sociale, Ministère du Travail et des Affaires Sociales 1995]. This low prevalence was thought to be the result of selection bias due to the inaccessibility of the population so soon after the war. A community based rehabilitation survey in Kigali estimated a prevalence of disability of 1.8% in 1997 [Jackson H.E. Prevalence of Disability Study, Christian Blind Mission, 2002]. There were concerns that this was an underestimate because political sensitivity may have caused people to withhold information about disabled members of their family. In contrast, the national census in 2002 estimated the prevalence of all disabilities at 4.8%[13]. It is because of this disparity in data that the Ministry of Health of Rwanda requested that a national survey be conducted to estimate the burden of MSI, to enable planning of future surgical and rehabilitation services.

The aim of this study was to develop a new tool to screen for and diagnose MSI and to develop and to pilot test the methodology for a national survey of MSI in Rwanda. The objectives of the national survey of MSI were:

1. To estimate the prevalence of MSI in Rwanda

2. To identify the causes of MSI in Rwanda

3. To estimate the coverage of MSI services in Rwanda

4. To assess the barriers to uptake of MSI services

5. To evaluate quality of life among people with MSI

## Methods

### Definition of musculoskeletal impairment

A definition of MSI was developed by taking as a starting point the standard ICF definition of impairment as "a loss or abnormality in body structure or physiological function". We made this specific to the musculoskeletal system, and we specifically included chronic pain, and added a minimum time duration of one month to exclude minor injuries and self limiting conditions. We also reviewed published literature and had consensus discussions with healthcare professionals managing MSI, including physiotherapists, rehabilitation technicians and doctors. The definition was then checked to make sure it covered all commonly diagnosed cases of MSI that were referred to existing services and were likely to be seen in the survey. The definition of MSI that we used was:

"a lack of normal structure or function, or an increase in pain or discomfort in the integument, muscles, bone or joints of the body of an individual, that has lasted at least one month and which limits function of the musculoskeletal system."

### Developing the survey tools

The survey tool used comprised two parts. Firstly a screening tool which was applied to everyone in the survey to identify suspect cases of MSI. Those who failed the screening tool (i.e. answered 'yes' to any of the first six questions in the screening tool, and had a condition that had lasted for at least one month or was permanent) were suspect cases of MSI and underwent the second part of the survey tool, a standardised examination protocol to confirm case status.

#### Development of the screening tool

To develop a screening tool for MSI we conducted a literature search to identify similar tools. Zaman's 'ten question questionnaire (TQQ)[14] was chosen as a starting point. The TQQ is a tool to screen for all types of disability that has been used in a number of studies [6,10]. We removed the questions that did not deal with physical impairment (e.g. 'Does your child appear to have difficulty in hearing?' and 'Compared with other children does the child have difficulty seeing, either in the daytime or at night?'). We directed the questions to the first person so that they could be used for an adult, and separated questions that asked for many concepts at once. Thus questions that asked about arms and legs together were separated into two questions. The screening tool was tested against the diagnoses to see if it could accurately identify them. Discussions were held with physiotherapists, paediatricians, orthopaedic surgeons, rehabilitation assistants and patients in focus groups in Malawi where the concepts of the tool were discussed and altered according to make the questions easy to understand. We then had a screening tool of 7 questions which asked participants about difficulties using the various parts of their musculoskeletal system difficulties with walking, whether they used a mobility aid, whether they felt they had any physical deformity, and the duration of their impairment (Table 1). Preparatory work was undertaken in Malawi as one of the authors (CL) was based there, and the pathology encountered was assumed to be similar to Rwanda.

It will be noted that question 6 in the screening tool asks about seizures and convulsions. From a purely scientific point of view these are not physical impairments and could have been excluded, but they were included in the protocol because in Rwanda patients with seizures often presented at physical disability facilities, and also because seizures are associated with other physical impairments such as burns and cerebral palsy. In future uses of this survey this component could easily be left out.

Tool validation was carried out in Malawi. Four rehabilitation technicians were trained to use the tool. 179 participants were screened, of whom 93 were identified by a gold standard as cases of MSI, and 86 were non-cases. The gold standard of who was or was not a case was determined by the assessment of an independent orthopaedic surgeon and physiotherapist, who were familiar with the definition of MSI. The rehabilitation technicians trained in the use of the screening questions and physical observations then independently applied the tool to all the cases and non cases. Intra-observer correlation, sensitivity, specificity and positive predictive value (PPV) were calculated.

#### Development of the standardised examination protocol

Individuals who failed the screening test (i.e. suspected cases of MSI) were further examined using the standardised examination protocol. It includes a structured physical examination testing key components of the musculoskeletal system, examining function (e.g. walking), as well as examining the affected part of the body to determine the nature of the impairment. It also includes a structured interview to gain a more detailed history of the musculoskeletal impairment. This section was developed to confirm and diagnose the MSI and was developed through consensus with health care professionals. It comprises 7 sections including:

*a. Examination: *The physiotherapists examined the case to help with determining the diagnoses and severity of the MSI. They carried out a structured examination where they asked the individual to carry out tasks which included walking and picking up small items with their thumb and index finger. They also examined the affected area. They were trained to examine musculoskeletal components of the affected region they were examining e.g. the range of motion of a joint, or the length of a limb.

*b. Diagnosis: *The standardised examination protocol includes an algorithmic classification system for diagnosis which allowed the non-medical health care professionals to identify potential diagnoses of the MSI without access to further investigation modalities. The algorithm led the physiotherapist to question what was the aetiological cause of the impairment and then within each large aetiological category, there were options of diagnosis which they further determined by examination and questioning of the case. The five main subgroups of diagnoses were defined as congenital, traumatic, infective, neurological and other acquired.(see Table 2) Up to two diagnoses were permissible per identified case of MSI. The diagnostic list was drawn up after consultation with in country health care professionals dealing with MSI over several years. The diagnoses can if wished be mapped onto ICD10 (International Classification of Diseases) codes, but this has not been done in Table 2 for simplicity, since each diagnoses includes a variety of different ICD10 codes.

*c. Area affected and nature of problem: *In the standardised examination protocol an examiner would also record information on the area of the body affected and the nature of the problem. The nature of the problem could include total absence, additional part, and deviating position (as outlined in ICF).

*d. Aetiology: *Where this was known it was recorded on the standardised examination protocol. It was determined by questioning that case about when the impairment developed and how it came about. Additional questions were posed to gain more information. For example if an infective cause was suspected the case would be questioned about systemic symptoms, or discharge from the area affected. The physiotherapists were trained as to what questions to ask for each aetiology available which included road traffic accidents, war, infection and familial.

*e. Severity: *Severity was determined using the parameters for the percentage of function outlined in the WHO reference book International Classification of Functioning (ICF)[15]. A loss of function of 5–24% was mild, 25–49% was moderate and 50–90% was severe.

*f. Quality of life : *The EQ-5D is a public domain quality of life questionnaire from the Euro-Qol group, which has been validated in a number of countries and cultural settings[16]. It allows the participant to indicate their health state by indicating the most applicable statement in five parameters, including mobility. The EQ-5D also includes a visual analogue scale (VAS) which is a self-scoring measure of current health status from 0 (worst imaginable health state) to 100 (best imaginable health state).

*g. Treatment: *The standardised examination protocol recorded treatment that had already been given and allowed the interviewer to assess treatment needed. Guidelines were given to the physiotherapist during training as to likely treatment modalities to consider for different diagnoses. So for example for talipes equinovarus it was suggested that up to age 1 physiotherapy and splinting alone could be considered, but above this age surgery would become more of a consideration depending on the function of the case.

*h. Barriers to treatment: *The standardised examination protocol recorded the patients own understanding of reasons why they may not have had treatment. These included services not being available, or the patient being unable to afford available services.

#### Translation

The tool was translated and back translated from English into Kinyarwandan by two medical translators, independently of each other.

#### Pilot study

The national survey of MSI was planned as an all age population based survey covering the whole of Rwanda, using the screening tool and standardised examination protocol to screen for and diagnose MSI.

Following training, a pilot survey of 680 individuals of all ages in nine clusters was carried out, prior to the national survey.

#### Sampling strategy

Six rural and three urban clusters were selected using a stratified probability-proportionate-to-size sampling strategy in two of the twelve provinces of Rwanda. A multistage stratified cluster random sampling with probability proportional to size procedure was chosen as the strategy for selection of a cross sectional, representative sample of the population of all ages. The primary sampling units (clusters) were the enumeration areas delineated in the national 2002 census. A list of enumeration areas was obtained from the government. Nine clusters were selected by probability proportional to size.

In each cluster a modified compact segment sampling method was used to identify the 80 people to be screened and examined. Maps were obtained for each of the selected clusters. The maps provided delineated "nyumbakumi" which were groups of ten households in the enumeration areas. (in Kinyarwandan 'nyumba' = house, and 'kumi' = ten) prior to the arrival of the screening team, trained personnel would visit the cluster and meet with the local administrative heads to determine changes to the local population and get information about the number of people in each of the nyumbakumi. Once they had updated the maps then the enumeration area was divided into segments of 80 people and one of the segments was chosen at random as the one to be sampled (e.g. if the population size of the enumeration area was 400 people then it would be divided into five segments). The segment would be informed of the need to be present on the day of examination. Eligible subjects were defined as residents of the household, that is, an individual who normally sleeps and eats their meals in that household for at least 6 months per year.

#### Survey data collection procedures (see Fig 1)

Screening and examination of individuals was undertaken in the household using the screening tool and standardised examination protocol described above. Each team consisted of one physiotherapist, one nurse and one driver. The team physiotherapist visited and screened every member of every household in a segment. Consent was taken, first from the head of the household and then from the individual, after explanation of the examination procedures. The screening test was applied for people aged 5 yrs and above, and for children less than 5 years the questions were answered by proxy by the child's main carer. those who failed the screening test (i.e. suspect cases) underwent the standardised examination protocol, along with a random 10% of people who passed the screening test (i.e. suspect non-cases). The supervisors checked items of all completed forms in the field. Non-responders were revisited throughout the day, but those who were finally unavailable were recorded as 'believed to have MSI' (with or without treatment) or 'believed not to have MSI', after consultation with family and community leaders.

#### Data entry

Data was double entered by data clerks onto a pre-prepared database in Epi-Data with consistency checks and comparative validation of double entered data. Analysis was performed in Stata 9.0.

#### Training of personnel

Two orthopaedic surgeons, two epidemiologists and a physiotherapist were responsible for training the survey team members concerning the screening, the enumeration methodology and the diagnostic process. Three physiotherapists, three nurses and three drivers made three teams. Other staff included two enumerators, four data processors, one epidemiological supervisor, one medical supervisor, one data processing supervisor and a field study leader. All supervisors accompanied the teams and worked as field supervisors on differing days

All participants of the survey team underwent training over a period of three weeks. The physiotherapists and nurses were trained on the screening tool and diagnosis. The enumerators were trained in the mapping and segmenting process. Inter observer validation was carried out by repeat examination by the physiotherapists of 59 individuals who were a mixture of cases and non-cases. Inter observer agreement was estimated between the physiotherapists with respect to correctly identifying a case and a non case, diagnosis, treatment needs and severity A detailed tool protocol manual was given which included information outlining responsibilities of team members, how to complete the tool and the enumeration processes.

#### Ethics

Ethical approval was obtained from the London School of Hygiene & Tropical Medicine, and the independent ethics committee in Rwanda. Permission to proceed was granted by the government and then consent had to be granted for each cluster. This involved getting the community leaders' stamped approval at province, district, sector and cell level for every cluster that we attended. Each participant gave verbal approval for examination, and written approval was taken for any photographs that were taken. All identified cases were referred to a central community rehabilitation centre where, medical members of the study team reviewed and referred them on for further treatment.

## Results

### Screening tool sensitivity and specificity

Of the 93 individuals identified as gold standard cases the 7 screening questions identified 92 of them as cases. Of the 86 individuals that were identified as gold standard non-cases the 6 screening questions identified all of them as non-cases. This gave the screening tool a sensitivity of 97.8% and sensitivity of 98.8% and a positive predictive value 99%.

### Interobserver agreement

Kappa agreement coefficients were calculated for the three physiotherapists. For agreement on whether an individual was a case or not the kappa coefficient was 0.92, for the diagnostic group 0.90, for the precise diagnosis 0.72, for severity of the case 0.59 and for treatment needed 0.49.

### Pilot study

680 eligible individuals were enumerated and 480 were screened, giving a response rate of 69%. Almost all the non-responders were unavailable (96%), and few refused to be examined (4%). The response rate was higher in rural areas (72%) then in urban areas (62%). The response rate varied by age group, as there was a lower response rate (62%) amongst the working age group of 15–50 years and higher in the 0 to 5 year age group (83%). The response rate was higher in females (75%) than in males (62%).

38 cases were identified by the screening tool, and only one individual was identified as a case by the screen that was subsequently not a case. The sample prevalence of MSI was 8.1% (95% CI = 5.6–10.6), and this was highest in peopled aged over 50s (18%, 95% CI 7.0 – 29.0; see Table 3)). 20 of the cases were female (52.6%). There were 45 diagnoses of MSI in 38 individuals with MSI (i.e. 7 people had two diagnoses). The most common sub group of diagnoses being trauma (39%), with 39% other acquired, 5% infective, 6% neurological and 1% congenital. The most common diagnoses were amputation (11%) and spine pain (11%). Other important diagnosis included fracture malunion and non-union (8.9%) and seizures (8.9%). The most common aetiological cause of MSI was trauma (see Table 4). Mild and moderate cases were each responsible for 42% of cases, and the remaining 16% of cases were severe.

11 of the MSI cases (29%) had not received any treatment and 89% needed at least one further modality of treatment (see Table 5). 15 cases (41%) needed surgery, while 75% needed physiotherapy. Of those that needed more treatment 24 (73%) said the reason they had not sought further treatment was because they could not afford it, while only 3 (9%) said it was because they did not feel the need for treatment.

For the responders health today the mean EQ-5D value for those who were examined without MSI was 66% (S.D. 23%) while for those with MSI it was 45% (S.D. 24%), giving a mean difference of 20% (95% CI 10–31).

## Discussion and conclusion

MSI is an important cause of morbidity and mortality worldwide [1]. It is likely that the burden of MSI is disproportionately larger in the developing world [4] but this is an area that has not been researched sufficiently. Estimates of the prevalence and causes of MSI are needed to plan services, make international comparisons and to monitor programs. For this to happen tools and methodology need to be developed that can measure the prevalence and causes of MSI that can be readily used in the resource-poor developing world.

The 7 question screening tool showed high sensitivity and specificity for identifying MSI, and a high positive predictive value. The high sensitivity and specificity continued into the pilot. High inter-observer agreement was obtained between the examiners in Rwanda, except for assigning treatment needed (kappa = 0.49). Because of this low kappa additional training was provided on treatment needs and options for MSIs.

Probability proportionate to size sampling worked well in the pilot study and an up-to-date sampling frame was available. For this study to have the security of a probability sampling method, we will use the compact segment sampling method for selecting secondary sampling units [18]. While this method requires increased skills in mapping, and is more complicated in its execution, it reduces the likelihood of selection bias that could arise if physiotherapists were allowed to select the households that they are to attend through the random walk method[18]. Compact segment sampling also facilitates call back at households where people were unavailable.

The pilot study demonstrated that the new screening and examination tool that was developed and the sampling methodology proposed for this study are appropriate and feasible for the purposes of this survey. The pilot study demonstrated that 80 people was the logistical limit for any team on one day, especially once travel times had been considered. The most important issue highlighted by the pilot for the main study was the response rate which was lower amongst the males in the study. The need for good participation of the community leaders was noted by the teams. In clusters where they were fully involved in both segmenting and on survey day there was a better response. Additionally the lower response rate in urban areas was often because people were at work and so during the main study the plan will be to carry out urban clusters later in the day and on weekends.

Overall this approach is appropriate for measuring the prevalence of MSI in Rwanda. Studies investigating the prevalence of conditions causing MSI are important steps to building sound epidemiological understanding of the nature of these disorders to enable appropriate public health planning.

## Competing interests

The author(s) declare that they have no competing interests.

## Authors' contributions

OA – collaborated in tool design, designed the methodology for the study, was part of the data acquisition team in the field, drafted the manuscript and has given final approval of the version to be submitted

HK – collaborated in designing the methodology for the study, helped draft and revised the manuscript and has given final approval of the version to be submitted

DR – made substantial contribution in data acquisition in the field, has been involved in revising the manuscript and has given final approval of the version to be submitted

VS – made substantial contribution in data acquisition in the field, has been involved in drafting the manuscript and has given final approval of the version to be submitted

CL – helped conceive the idea for the study, collaborated on tool design, revised the manuscript and has given final approval of the version to be submitted.

All authors have read and approved the final manuscript

## Pre-publication history

The pre-publication history for this paper can be accessed here:



## Figures and Tables

**Figure 1:**
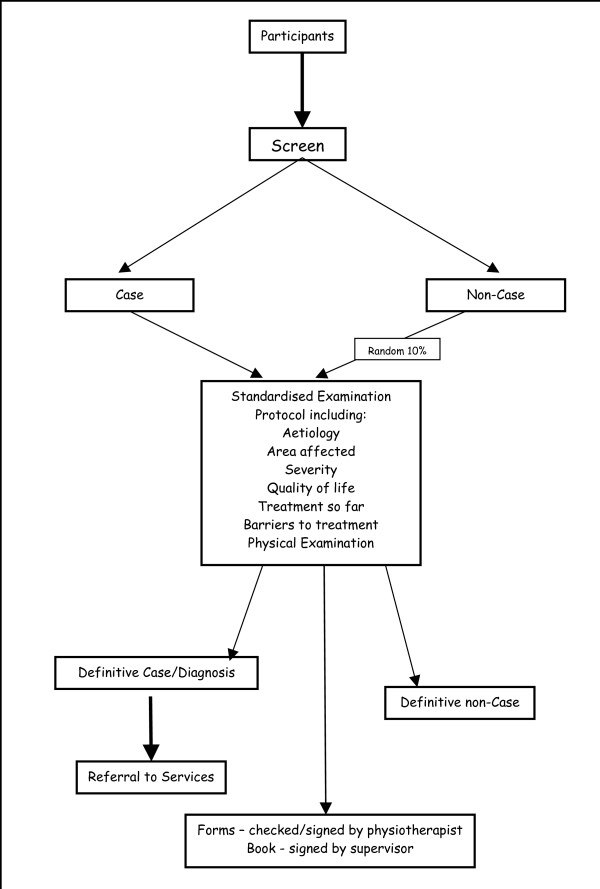
Data Collection Procedures.

**Table 1:** Screening questionnaire

Screen for musculoskeletal impairment	Yes	No
1. Is any part of your body missing or misshapen?	○	○
2. Do you have any difficulty using your arms?	○	○
3. Do you have any difficulty using your legs?	○	○
4. Do you have any difficulty using any other part of your body?	○	○
5. Do you need a mobility aid or prosthesis?	○	○
6. Do you have convulsions, involuntary movement, rigidity or loss of consciousness?	○	○
		
If any of the answers are "yes"		
7. Has it lasted more than one month or is it permanent?	○	○

**Table 2:** diagnostic criteria for the survey

	Diagnosis	Definition
**A**	**Congenital deformity**	
A1	Polydactyly	More than five digits on one or both hands (can also be on the feet)
A2	Syndactyly	Digits joined together by skin or soft tissue (can also be on the feet)
A3	Other upper limb deformity	Any congenital abnormality of upper limb or foot not already described
A4	Club foot	Eqinus and varus deformity of foot which is not passively correctable
A5	Other lower limb deformity	Any congenital abnormality of lower limb or foot not already described
A6	Spine deformity	Any congenital deformity of spine
A7	Cleft lip/palate	Unilateral or bilateral defect in continuity of upper lip, with or without associated defect in palate
A8	Multiple abnormalities	Congenital abnormalities in more than one site, not including polydactyly
A9	Other – specify	Other congenital abnormality not described above
		
**B**	**Trauma**	
B1	Fracture non/malunion	Un-united fracture more than six months since injury or fracture healed with deformity e.g. Shortening or angulation
B2	Burn contracture	Burn scar of skin or soft tissue causing deformity
B3	Spine injury	Spinal injury resulting in deformity and/or neurological deficit
B4	Head injury	Head injury resulting in neurological
B5	Joint chronic dislocation	Joint dislocation that remains unreduced or regularly re-dislocates
B6	Other chronic joint injury	Pain or stiffness or swelling or other joint symptom following trauma, but not including dislocation
B7	Tendon/muscle/nerve injury	Tendon or muscle or soft tissue or nerve injury or pain following trauma
B8	Amputation	Loss of all or part of a limb due to accident or surgery
B9	Other – specify	Any other traumatic injury not previously described
		
**C**	**Infective**	
C1	Joint infection	Loss of function of joint associated with history of swelling and fever or discharge
C2	Bone infection limb	Pain and or deformity in limb associated with fever, or abscess or discharge of pus
C3	Bone infection spine	Pain and or deformity in spine associated with fever, or abscess or discharge of pus
C4	Skin/soft tissue infection/wound	Infection of skin or soft tissue including wound infection and ulcers
C5	Other – specify	Any other infection affecting the musculoskeletal system not described above
		
**D**	**Neurological**	
D1	Epilepsy/seizures	2 or more seizures/convulsions not associated with fever
D2	Leprosy	Known diagnosis of leprosy
D3	Polio	Known polio infection
D4	Para/quadri/tetraplegia	Marked weakness or paralysis in arms or legs or both, may be due to injury or other causes
D5	Cp/developmental delay	Delay in attaining normal childhood milestones with not obvious physical disorder
D6	Cerebral palsy	Nonprogressive motor deficit which started before age 10
D7	Peripheral nerve palsy	Weakness due to single nerve lesion e.g. Unilateral foot drop or wrist drop
D8	Other – specify	Sensory or motor deficit or neurological disorder not already described
		
**E**	**Other acquired**	
E1	Joint problem	Joint pain or swelling or deformity with no recent preceding trauma
E2	Angular limb deformity	Abnormal angulation of limb at or near joint. Kncluded knock knees and bow legs
E3	Bone tumour/swelling	Firm swelling of bone – benign or malignant
E4	Skin/soft tissue tumour/swelling	Swelling of soft tissue – benign or malignant
E5	Spine deformity	Acquired deformity of spine
E6	Spine pain	Spinal pain limiting function but not associated with significant deformity
E7	Limb pain	Limb pain limiting function, but no structural change
E8	Limb swelling	Abnormal swelling of limb (includes lymphoedema)
E9	Other – specify	Aquired musculosekeletal impairment not due to infection or trauma, not neurological and not described above

**Table 3:** Number of cases and MSI prevalence by age group

Age	Number of cases (%)	Number of people screened (%)	Prevalence(%) of MSI by age group (95% CI)
0 to 5	6 (15.8)	94 (20.1)	6.4 (1.3 – 11.4)
6 to 14	4 (10.5)	99 (21.2)	4.0 (0.1 – 8.0)
15 to 50	19 50.0)	225 (48.0)	8.5 (4.8–12.2)
Over 50	9 (23.7)	50 (10.7)	18.0 (7.0 – 290)
Total	38 (100.00)	468 (100.0)	8.1 (5.6 – 10.6)

**Table 4:** Causes of MSI by aetiology

	Number	Prevalence	% of cases (n = 45)	Confidence limits
Family history	3	0.6	6.7	0.1–1.9
Congenital, no family history	2	0.4	4.4	0.1–1.5
Perinatal hypoxia	1	0.2	2.2	0.03–0.8
Trauma	16	3.4	35.6	2.0–5.5
Infection	4	0.9	8.9	0.2–2.2
Neoplasm	1	0.2	2.2	0.03–0.8
Unknown	17	3.6	37.8	2.1–5.8
Other	1	0.2	2.2	0.03–0.8
Total (no of diagnoses)	45		100.0	

**Table 5:** cases by diagnosis and diagnostic subgroups showing treatment needs

Diagnostic group		Diagnosis	N (%)	Proportion needing further treatment (%)
A congenital	A3	Other congenital deformity of upper limb	1	100
B traumatic	B1	Fracture Mal/Nonunion	4	100
	B2	Burn Contracture	1	100
	B6	Other chronic joint injury	3	100
	B7	Tendon Problem	1	0
	B8	Amputation	5	60
	B9	Other Trauma	2	50
C infective	C2	Bone Infection	1	100
	C4	Soft tissue infection/cellulites/skin wound	1	100
D neurological	D1	Seizures	4	100
	D6	Cerebral Palsy	1	100
	D7	Peripheral Nerve Palsy	1	100
	D8	Other neurological	1	100
E acquired	E1	Joint Problem	3	100
	E2	Knock Knees	2	100
	E5	Spine deformity	1	100
	E6	Spine Pain	5	100
	E8	Limb Swelling	1	100
	E9	Other acquired non traumatic	7	100
